# 

**Published:** 1908-08

**Authors:** 


Sixty-fourth Year.

VOL. LXIV. AUGUST, 1908. No. 1

### BUFFALO MEDICAL JOURNAL

LIBRARY
Indexed
S. G. O.

Established 1845 by AUSTIN FLINT M. D.

EDITOR:

WILLIAM WARREN POTTER, M. D.

ASSISTANT EDITORS:

WILLIAM C. KRAUSS, M. D. NELSON W. WILSON, M. D.

ASSOCIATE EDITORS:

JAMES WRIGHT PUTNAM, M. D. ERNEST WENDE, M. D.
JOHN PARMENTER, M. D. JOHN A. MILLER, PH. D.
MAUD J. FRYE, M. D.

